# Herd Immunity to Ebolaviruses Is Not a Realistic Target for Current Vaccination Strategies

**DOI:** 10.3389/fimmu.2018.01025

**Published:** 2018-05-09

**Authors:** Stuart G. Masterson, Leslie Lobel, Miles W. Carroll, Mark N. Wass, Martin Michaelis

**Affiliations:** ^1^Industrial Biotechnology Centre and School of Biosciences, University of Kent, Canterbury, United Kingdom; ^2^Department of Microbiology, Immunology and Genetics, Faculty of Health Sciences, Ben-Gurion University of the Negev, Beer-Sheva, Israel; ^3^Department of Emerging and Re-Emerging Diseases and Special Pathogens, Uganda Virus Research Institute (UVRI), Entebbe, Uganda; ^4^Research & Development Institute, National Infection Service, Public Health England, Porton Down, Salisbury, United Kingdom

**Keywords:** ebola virus, Ebolavirus, vaccines, herd immunity, basic reproduction number

## Abstract

The recent West African Ebola virus pandemic, which affected >28,000 individuals increased interest in anti-Ebolavirus vaccination programs. Here, we systematically analyzed the requirements for a prophylactic vaccination program based on the basic reproductive number (*R*_0_, i.e., the number of secondary cases that result from an individual infection). Published *R*_0_ values were determined by systematic literature research and ranged from 0.37 to 20. *R*_0_s ≥ 4 realistically reflected the critical early outbreak phases and superspreading events. Based on the *R*_0_, the herd immunity threshold (*I*_c_) was calculated using the equation *I*_c_ = 1 − (1/*R*_0_). The critical vaccination coverage (*V*_c_) needed to provide herd immunity was determined by including the vaccine effectiveness (*E*) using the equation *V*_c_ = *I*_c_/*E*. At an *R*_0_ of 4, the *I*_c_ is 75% and at an *E* of 90%, more than 80% of a population need to be vaccinated to establish herd immunity. Such vaccination rates are currently unrealistic because of resistance against vaccinations, financial/logistical challenges, and a lack of vaccines that provide long-term protection against all human-pathogenic Ebolaviruses. Hence, outbreak management will for the foreseeable future depend on surveillance and case isolation. Clinical vaccine candidates are only available for Ebola viruses. Their use will need to be focused on health-care workers, potentially in combination with ring vaccination approaches.

## Introduction

The genus *Ebolavirus* contains five species: *Zaire ebolavirus* (type virus: Ebola virus), *Sudan ebolavirus* (type virus: Sudan virus), *Bundibugyo ebolavirus* (type virus: Bundibugyo virus), *Taï Forest ebolavirus* (type virus: Taï Forest virus, previously also referred to by names such as Côte d’Ivoire ebolavirus or Ivory Coast ebolavirus), *Reston ebolavirus* (type virus: Reston virus) (1). Four Ebolaviruses (Ebola virus, Sudan virus, Bundibugyo virus, Taï Forrest virus) are endemic to Africa and can cause severe disease in humans (2). Reston viruses are endemic to Asia and considered to be non-pathogenic in humans (2). However, very few genetic changes may result in human-pathogenic Reston viruses (2–4). Since the discovery of the first two members of the *Ebolavirus* family in 1976 in Sudan (today South Sudan) and Zaïre (today Democratic Republic of Congo), Ebolaviruses had until 2013 only caused small outbreaks in humans affecting up to a few 100 individuals (5, 6). The recent Ebola virus outbreak in West Africa (2013–2016) resulted in 28,616 confirmed, probable, and suspected cases of Ebola virus disease and 11,310 deaths (6), which may still underestimate the actual numbers (7). It was the first Ebolavirus outbreak that affected multiple countries, was introduced to another country *via* air travel, and resulted in a significant number of human disease cases outside of Africa (5, 6). Prior to this outbreak, only isolated human cases were treated outside of Africa. A scientist who had become infected by Taï Forest virus after an autopsy of a Chimpanzee was treated in Switzerland (8), and two laboratory infections were reported in Russia (9, 10). In addition, Reston virus-infected non-human primates were exported from the Philippines to the US and Italy (11). Finally, Marburg virus (which belongs like the Ebolaviruses to the Filoviruses) was exported out of Africa (12, 13) and was associated with laboratory infections (14, 15). Due to its unique size, the West African Ebolavirus outbreak emphasized the health threats posed by Ebolaviruses and the importance of protection strategies (6, 7).

Vaccination programs are effective in controlling infectious diseases, as demonstrated by the WHO-driven smallpox eradication (16). However, eradication is likely to be more difficult for zoonotic viruses like the Ebolaviruses that circulate in animal reservoirs (17). Only herd immunity could prevent future outbreaks and protect individuals that cannot be vaccinated due to health issues (16). The herd immunity threshold (*l*_c_) describes the number of society members that need to be protected (18) to prevent outbreaks. It is based on the basic reproductive number *R*_0_ (number of secondary cases caused per primary case) of a pathogen (18–22).

Here, we performed a systematic analysis to determine the critical vaccine coverage (*V*_c_) required to prevent Ebolavirus outbreaks by a prophylactic mass vaccination program based on the *R*_0_ associated with Ebolavirus infection in humans. The results were further critically considered in the context of (1) the status of current Ebolavirus vaccine candidates and (2) the feasibility of a large-scale prophylactic Ebolavirus vaccination program taking into account (a) the preparedness to participate in vaccination programs in the affected societies, (b) logistic challenges, and (c) costs.

## Materials and Methods

### Identification of Studies That Report on the Basic Reproductive Number (*R*_0_) of Ebolaviruses

To identify scientific articles that have calculated the basic reproductive number (*R*_0_) for Ebolaviruses, we performed a literature search using PubMed (www.ncbi.nlm.nih.gov/pubmed) for the search term combinations “Ebola R0,” “Ebola basic reproductive number,” and “Ebola basic reproduction number” (retrieved on 29th September 2017).

### Determination of Herd Immunity Thresholds and Their Implications for Ebolavirus Diseases Prevention Strategies

Based on the basic reproductive number *R*_0_, i.e., the number of secondary cases that result from an individual infection, the herd immunity threshold (*I*_c_) was calculated using Eq. 1.

(1) $$I_{\text{c}}=1-\left(1/R_{0}\right)$$

where *I*_c_ indicates the proportion of a society that needs to be protected from infection to achieve herd immunity. Next, the critical vaccination coverage (*V*_c_) that is needed to provide herd immunity was determined by including the vaccine effectiveness (*E*) using Eq. 2 (18–22).

(2) $$V_{\text{c}}=I_{\text{c}}/E=\left[1-\left(1/R_{0}\right)\right]/E$$

## Results

### Basic Reproductive Number (*R*_0_) Values for Ebolaviruses

**Figure 1:**
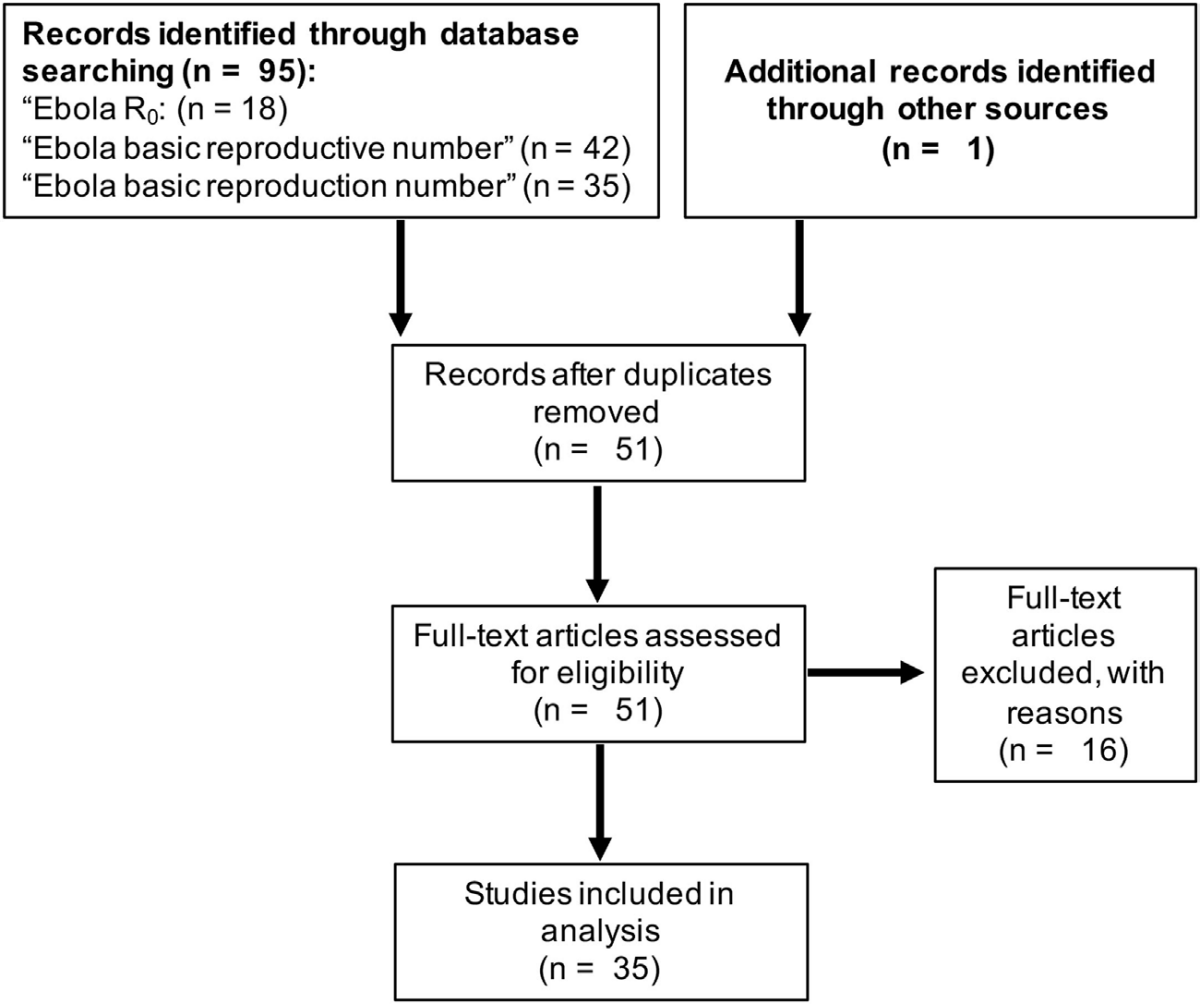
Summary of the literature search using PubMed (www.ncbi.nlm.nih.gov/pubmed) to identify articles that report on the basic reproductive number (*R*_0_) of Ebolaviruses.

The PubMed search for “Ebola R0” provided 18 hits, the search for “Ebola basic reproductive number” provided 42 hits, and the search for “Ebola basic reproduction number” provided 35 hits (Figure 1; Data Sheet S1 in Supplementary Material). After removal of the overlaps and inclusion of an additional article [identified from the reference list of Ref. (21)], this resulted in 51 articles, 35 of which provided relevant information on Ebolavirus *R*_0_ values (Figure 1; Data Sheet S1 in Supplementary Material).

*R*_0_ data were only available for Ebola virus and Sudan virus outbreaks (Data Sheet S1 in Supplementary Material). 29/35 studies analyzed data from the recent West African Ebola virus outbreak (Data Sheet S1 in Supplementary Material). The others reported on Ebola virus outbreaks in the Democratic Republic of Congo. Four studies also included data from the Sudan virus outbreak 2000/2001 in Gulu, Uganda. We also considered a review that summarized all available data until February 2015 (5) (Data Sheet S1 in Supplementary Material).

*R*_0_ indicates the number of new infections caused by an infected individual, and when greater than 1, an outbreak will spread. Different approaches to calculate *R*_0_s lead to varying results (22). Accordantly, *R*_0_ values calculated for the Sudan virus outbreak 2000/2001 in Gulu using identical data ranged from 1.34 to 3.54 (Data Sheets S1 and S2 in Supplementary Material). Small outbreak sizes may also limit the accuracy of the calculated *R*_0_ values. Additionally, virus transmission is influenced by socioeconomic and behavioral factors including the health-care response, society perceptions, religious practices, population density, and/or infrastructure (22, 23). Concordantly, *R*_0_s that were determined by the same methodology in different districts of Guinea, Liberia, and Sierra Leone during the West African Ebola virus epidemic ranged from 0.36 to 3.37 (24). Three studies directly compared the Ebola virus outbreak in Kikwit (1995, DR Congo) and the Sudan virus outbreak in Gulu (2000/2001, Uganda) (25–27), but did not reveal fundamental differences between the *R*_0_s of the viruses (Data Sheets S1 and S2 in Supplementary Material). Across all relevant studies, *R*_0_s ranged from 0.36 to 12 for Ebola virus and from 1.34 to 3.54 for Sudan virus (Data Sheet S1 in Supplementary Material). 9 of the 35 studies that provided *R*_0_ values showed that Ebola viruses can spread with an *R*_0_ > 3, and five studies suggested that Ebolaviruses can spread with *R*_0_ values > 4. High reproductive numbers (≥4) are typically observed at the beginning of Ebolavirus outbreaks, prior to the implementation of control measures (28–31). Also, the spread of Ebolaviruses may be substantially driven by “superspreaders” who infect a high number (up to 15–20) of individuals (23, 32–35). Studies from the West African Ebola virus outbreak suggested that relatively small numbers of superspreaders may have been responsible for the majority of cases (35, 36). Since the available data suggest that Ebolavirus transmission can occur with *R*_0_ values of 3, 4, or even higher, a prophylactic vaccination program should establish herd immunity against Ebolaviruses that spread at such levels.

### Herd Immunity Threshold (*I*_c_)

**Figure 2:**
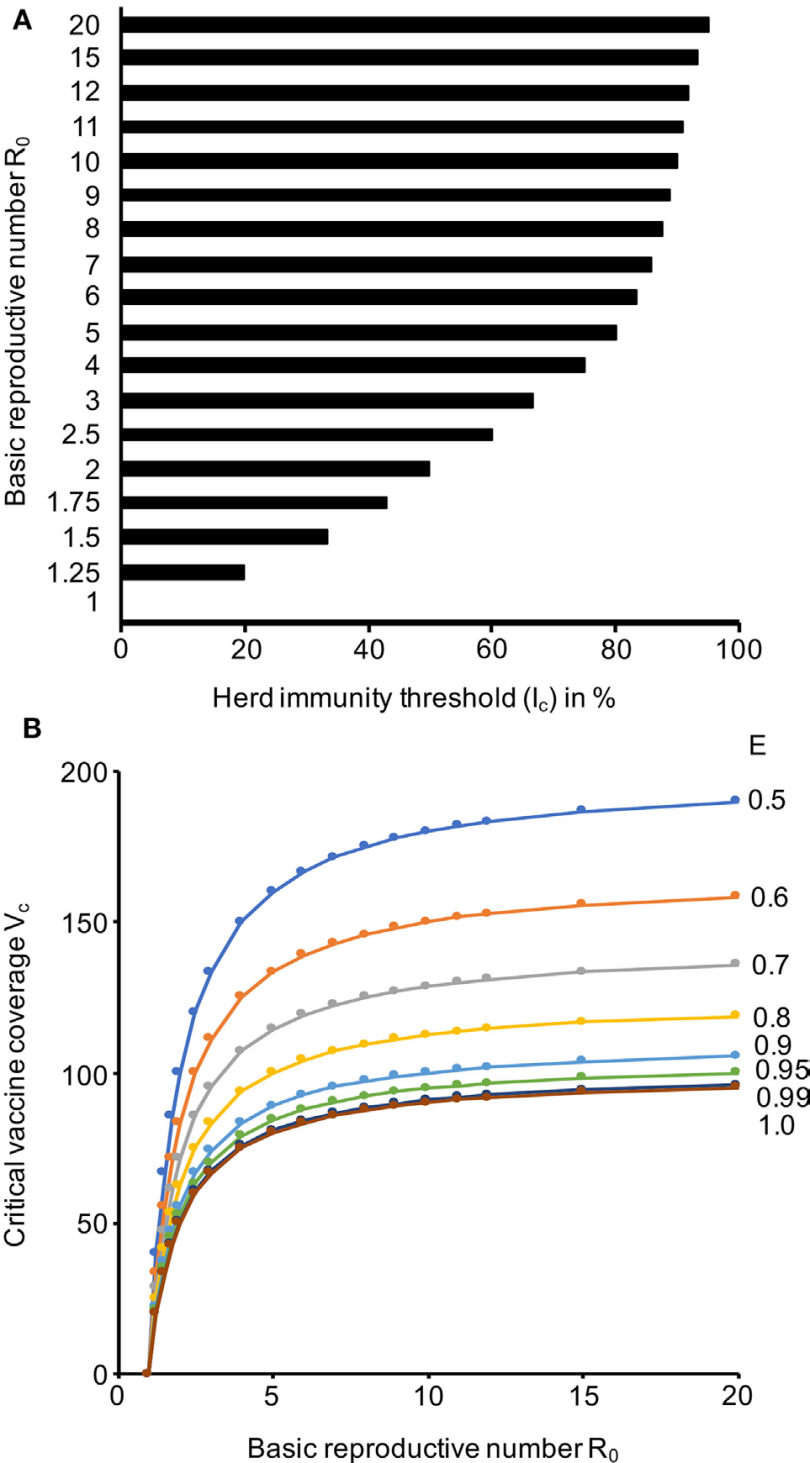
Herd immunity thresholds (*I*_c_) and critical vaccine coverage (*V*_c_) values in dependence of the basic reproductive number (*R*_0_) and the vaccine efficacy (*E*). **(A)** *I*_c_ values based on a range of *R*_0_ values that cover the range reported for Ebola viruses. **(B)** *V*_c_ values based on *R*_0_ values that cover the range reported for Ebola viruses and *E* values that are in the range of those reported for approved vaccines. The respective numerical data are presented in Data Sheet S3 in Supplementary Material.

At an *R*_0_ of 3, the *I*_c_ (Eq. 1) is 67%, which means that 67% of a population need to be immune to provide herd immunity (Figure 2A; Data Sheet S3 in Supplementary Material). The *I*_c_ further rises to 75% at an *R*_0_ of 4, to 80% at an *R*_0_ of 5, to 90% at an *R*_0_ of 10, and to 95% *R*_0_ of 20 (Figure 2A; Data Sheet S3 in Supplementary Material). This shows that high proportions of a population need to be immune to establish effective herd immunity.

### Critical Vaccine Coverage (*V*_c_)

As there is currently no approved vaccine for the prevention of Ebolavirus disease, we calculated a range of *V*_c_ (Eq. 2) scenarios that reflect the efficacy range covered by approved vaccines. Attenuated replication-competent measles virus vaccines have been reported to protect up to 95% of individuals from disease after one dose, which increased to up to 99% after a second dose (37). The efficacy of varicella zoster virus vaccines, another attenuated replication-competent vaccine, was recently calculated to be 81.9% after one dose and 94.4% after two doses (38). Inactivated seasonal influenza virus split vaccines have been reported to have a substantially lower efficiency of 50–60% (39–41). Hence, we considered a *V*_c_ range between 50 and 100% (Figure 2B; Data Sheet S3 in Supplementary Material). Vaccines, which provide high protection (ideally after a single vaccination), and high vaccination rates are required for prophylactic vaccination programs that establish a level of herd immunity that prevents Ebolavirus outbreaks. If we assume an *R*_0_ of 3 and a vaccination efficacy *E* of 90%, more than 70% of a population need to be vaccinated to establish herd immunity. At an *R*_0_ of 4 and a vaccination efficacy *E* of 90%, more than 80% of a population need to be vaccinated. If the *R*_0_ rises to 5, a vaccine coverage of 80% would be required, even if a vaccine with 100% efficacy was available (Figure 2B; Data Sheet S3 in Supplementary Material).

## Discussion

We performed an analysis of the Ebolavirus vaccine requirements to achieve the *V*_c_ needed for prophylactic mass vaccination programs. A number of studies suggested that Ebolavirus transmission can occur with *R*_0_ values of 3, 4, or even higher, in particular during early outbreak stages (prior to the implementation of control measures) and/or as consequence of superspreading events (23, 24, 28–36). Therefore, a prophylactic vaccination program should establish herd immunity against Ebolaviruses that spread at such levels. At an *R*_0_ of 3, >70% of individuals and at an *R*_0_ of 4, >80% of individuals need to be vaccinated with a vaccination efficacy of 90% to achieve herd immunity. Hence, highly effective vaccines and a high vaccination coverage are essential for successful prophylactic mass vaccination programs against Ebolaviruses.

Clinical vaccine candidates providing protection against all three to four human-pathogenic Ebolaviruses (Ebola virus, Sudan virus, Bundibugyo virus, potentially Taï Forest virus) do not currently exist (Data Sheet S4 in Supplementary Material), although preclinical data suggest that the development of such vaccines may be feasible (6). Current vaccine candidates may also not provide the long-term protective immunity (≥10 years) necessary for sustainable protection against spillover events from animal reservoirs. Two studies reported immune responses 12 months after vaccination with different Ebola virus vaccine candidates (42, 43). One of them described seroconversion in >90% of individuals after a single injection of rVSV-ZEBOV, a vesicular stomatitis virus-based Ebola virus vaccine. No or only a minor drop in antibody titers and neutralization capacity was reported 360 days after vaccination (42). A study investigating rVSV-ZEBOV and ChAd3-EBO-Z, a chimpanzee adenovirus type-3 vector-based Ebola virus vaccine, found lower seroconversion rates (rVSV-ZEBOV: 83.7%; ChAd3-EBO-Z: 70.8%) and reported the highest antibody response after 1 month and a decline afterward (43). Thus, it is not clear, whether the vaccine-induced immunity covers the time frame of 2 years (or perhaps even longer) that Ebolavirus survivors may remain contagious for (6, 42–52). It is also not clear whether (and if yes, to which extent) immunity to Ebolaviruses is mediated by cell-mediated and/or humoral immune responses (53). A challenge study using non-human primates suggested that protection by adenovirus-based vaccines is cell mediated (54). This means that antigen binding and/or neutralization titers may not always correlate with protection from disease. Consequently, the efficacy levels of vaccines cannot be determined with certainty based on antibody responses at various time points post vaccination. Thus, it remains unknown whether current vaccine candidates offer the long-term protection necessary for mass vaccination programs that effectively prevent zoonotic Ebolavirus outbreaks. Ebola virus recurrences and reinfections indicate that, although natural Ebolavirus infections are generally assumed to provide long-term protection, natural infections may not always result in sustained protective immunity in every survivor, which may further complicate the development of vaccines that provide long-term protection (55, 56). In this context, the establishment of long-term immunity may be influenced by the disease treatment. In a case of relapse 9 months after discharge, it was speculated whether the treatment of the initial disease with convalescent plasma and monoclonal antibodies might have contributed to the recurrence (55).

Limited acceptance of vaccinations may also limit Ebolavirus vaccination programs. In a rVSV-ZEBOV ring vaccination trial, only 5,837/11,841 patient contacts could be vaccinated. 34% of the contacts refused the vaccination (57). In a survey in Sierra Leone during the West African Ebola epidemic, 106/400 respondents (26.6%) were prepared to pay for a vaccination, while 290 respondents (72.5%) would have accepted a free vaccination (58). Since 74% of the population need to be vaccinated by a vaccine with a 90% efficacy to prevent an outbreak that spreads with an *R*_0_ of 3 and 83% of the population to prevent an outbreak that spreads with an *R*_0_ of 4 (Data Sheet S3 in Supplementary Material), such levels of vaccine coverage seem currently unachievable, even under the threat of an ongoing epidemic, although attitudes may change in the future if more (clinical) data becomes available. Therefore, more differentiated vaccination strategies with a focus on health-care workers and patient contacts appear more feasible.

The median maximum fee that survey participants in Sierra Leone during the West African Ebola epidemic were prepared to pay for a vaccine was about 5,000 leones ($0.65 as of 11th January 2018) (58). The international organization GAVI (www.gavi.org) is providing $5 million for the development of rVSV-ZEBOV, which is expected to pay for 300,000 vaccine doses (about $16.70/dose) (59). Within a rVSV-ZEBOV ring vaccination trial, 11,841 contacts requiring vaccination from 117 clusters were identified over a 10-month period, i.e., about 101 individuals per confirmed Ebola virus disease patient (57). Hence, 300,000 doses will enable vaccination of the contacts of approximately 2,970 Ebola virus disease patients. If an effective vaccine (which provided protection against all human-pathogenic Ebolaviruses) was available, a vaccination program would comprise about 462 million individuals in the countries that have been affected by Ebolavirus outbreaks (Data Sheet S5 in Supplementary Material). Notably, the countries, which have been affected by Ebolavirus outbreaks so far, have large rural populations ranging from 13% (Gabon) to 84% (Uganda) (Data Sheet S5 in Supplementary Material). Vaccination programs in rural areas are associated with logistical issues including transport difficulties, lack of equipment and trained medical specialists, and cultural and language barriers (60, 61).

In conclusion, the achievement of a *V*_c_ of 75% that is necessary to prevent an outbreak that spreads with an *R*_0_ of 4 with a vaccine that has an efficacy of 100% is currently unrealistic because of limited vaccine acceptance in the affected populations and because of financial and logistical challenges. In addition, concurrent diseases such as HIV and cancer, along with potential side effects of vaccination, may remove significant numbers of potential vaccines (6, 62). Alternative vaccination strategies will be required for such patients. Replication-deficient vaccines such as DNA vaccines, virus-like particles, nanoparticle-based vaccines, and viral vectors (e.g., Modified Vaccinia Ankara, which was already demonstrated to be safe in immunocompromised individuals) may be safer alternatives (6, 63). Moreover, vaccines that provide long-term immunity against all three (or including Taï Forest virus, four) human-pathogenic Ebolaviruses, which would be needed to protect populations effectively from large Ebolavirus outbreaks in endemic areas, do not exist. Therefore, outbreak control of Ebolaviruses will for the foreseeable future depend on surveillance and the isolation of cases. Clinical vaccine candidates are only available for Ebola viruses and will need to be focused on health-care workers, who are often involved in disease transmission (30), potentially in combination with the vaccination of patient contacts. Hence, our findings support the conclusions of the WHO Strategic Advisory Group of Experts on immunization (SAGE) at the WHO SAGE meeting on 25th to 27th April 2017 (64). SAGE acknowledged the need for further research on Ebolavirus vaccines, including the generation of conclusive data on the duration of protection provided by Ebolavirus vaccine candidates. In case of future Ebolavirus outbreaks, SAGE recommended the use of rVSV-ZEBOV ring vaccination strategies (64).

## Author Contributions

SM performed the calculations. MM performed the literature search. All authors analyzed the data. SM, MM, and MW wrote the manuscript. All authors gave their final approval of the version to be published.

## Conflict of Interest Statement

The authors declare that the research was conducted in the absence of any commercial or financial relationships that could be construed as a potential conflict of interest.
