# Supplementary material for: Herd Immunity to Ebolaviruses Is Not a Realistic Target for Current Vaccination Strategies
Source: Front Immunol. 2018 May 9;9:1025. doi: 10.3389/fimmu.2018.01025 (PMC5954026; doi:10.3389/fimmu.2018.01025)
Supplement: Supplementary file 2 [file Data_Sheet_2.PDF]

Data Sheet 2

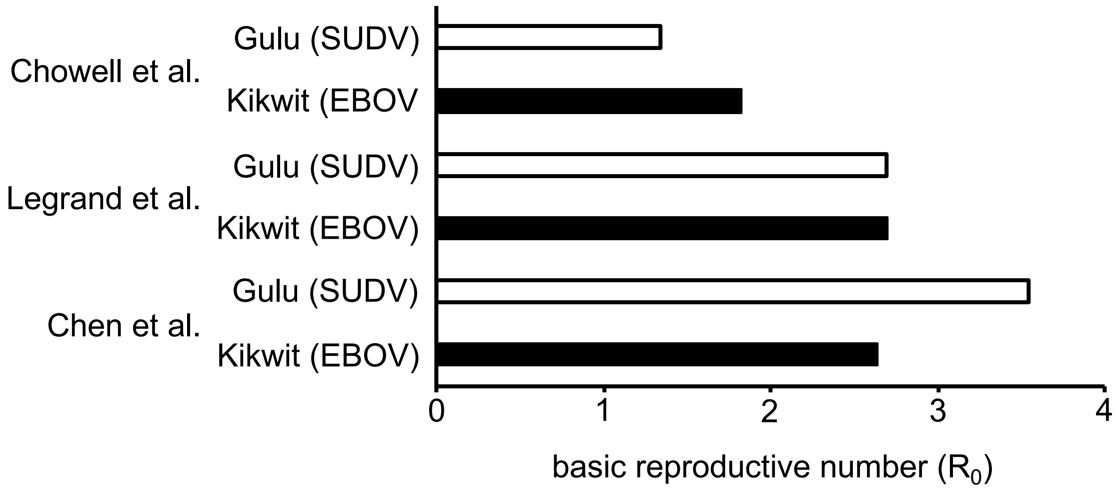

Data Sheet 2. Basic reproductive numbers (R_0) determined for the Ebola virus outbreak in Kikwit (DR Congo, 1995) and the Sudan virus outbreak in Gulu (Uganda, 2000/ 2001) as determined in three different studies (15-17).
