# Supplementary material for: Herd Immunity to Ebolaviruses Is Not a Realistic Target for Current Vaccination Strategies
Source: Front Immunol. 2018 May 9;9:1025. doi: 10.3389/fimmu.2018.01025 (PMC5954026; doi:10.3389/fimmu.2018.01025)
Supplement: Supplementary file 3 [file Data_Sheet_3.PDF]

Data Sheet 3. Herd immunity thresholds (I_c) values based on a range of basic reproductive number (R_0) values that cover the range reported for Ebolaviruses (numerical data underlying Figure 3A).

R_0	$I_c = 1 - (1/R_0)$
1	0
1.25	20.0
1.5	33.3
1.75	42.9
2	50.0
2.5	60.0
3	66. 7
4	75.0
5	80.0
6	83.3
7	85.7
8	87.5
9	88. 9
10	90.0
11	90.9
12	91. 7
15	93.3
20	95.0

Critical vaccine coverage V_c values based on basic reproductive number (R_0) values that cover the range reported for Ebolaviruses and vaccine efficacies (E) that are in the range of those reported for approved vaccines (numerical data underlying Figure 3B).

	$V_c = I_c/E$							
R_0	E = 0.5	E = 0.6	E = 0.7	E = 0.8	E = 0.9	E = 0.95	E = 0.99	E = 1.0
1	0	0	0	0	0	0	0	0
1.25	40.0	33.3	28.6	25	22.2	21.1	20.2	20.0
1.5	66. 7	55. 6	47.6	41. 7	37.0	35.1	33. 7	33.3
1.75	85.7	71.4	61.2	53.6	47.6	45.1	43.3	42.9
2	100	83.3	71.4	62.5	55. 6	52.6	50.5	50.0
2.5	120	100	85.7	75.0	66. 7	63.2	60.6	60.0
3	133	111	95.2	83.3	74.1	70.2	67.3	66. 7
4	150	125	107	93.8	83.3	78.9	75.8	75.0
5	160	133	114	100.0	88. 9	84.2	80.8	80.0
6	167	1389	119	104	92.6	87.7	84.2	83.3
7	171	143	122	107	95.2	90.2	86.6	85.7
8	175	146	125	109	97.2	92.1	88.4	87.5
9	178	148	127	111	98.8	93. 6	89.8	88. 9
10	180	150	129	113	100	94.7	90.9	90.0
11	182	152	130	114	101	95.7	91.8	90.9
12	183	153	131	115	102	96.5	92.6	91. 7
15	187	156	133	117	104	98.2	94.3	93.3
20	190	158	136	119	106	100	96.0	95.0
