# Supplementary material for: Herd Immunity to Ebolaviruses Is Not a Realistic Target for Current Vaccination Strategies
Source: Front Immunol. 2018 May 9;9:1025. doi: 10.3389/fimmu.2018.01025 (PMC5954026; doi:10.3389/fimmu.2018.01025)
Supplement: Supplementary file 4 [file Data_Sheet_4.PDF]

Data Sheet 4. Ebolavirus vaccine candidates in clinical testing (Keshwara et al., Ledgerwood et al.)

Vaccine candidate	Clinical phase
rVSV-ZEBOV (recombinant attenuated replication-competent vesicular stomatitis virus vector expressing Ebola virus GP)	Phase 3
Recombinant replication-deficient adenovirus serotype 5 (rAd5) vector expressing Ebola virus and Sudan virus GP	Phase 2
Recombinant replication-deficient adenovirus serotype 5 (rAd5) vector expressing GP from the 2014 Ebola virus outbreak in Guinea	Phase 1/2 (approved in China)
ChAd3-EBO-Z (recombinant replication-deficient chimpanzee adenovirus 3 vector expressing Ebola virus GP)	Phase 1/2
Ad26-EBOV (human recombinant adenovirus 26 vector expressing Ebola virus GP) / MVA-BN Filo (replication-deficient modified vaccinia Ankara expressing Ebola virus GP, Sudan virus GP, Marburg virus GP, and Taï Forest virus NP) boost	Phase 1

Keshwara R, Johnson RF, Schnell MJ. Toward an effective Ebola virus vaccine. Annu Rev Med (2017) 68:371–86. doi:10.1146/annurev-med-051215-030919

Ledgerwood JE, Costner P, Desai N, Holman L, Enama ME, Yamshchikov G, et al. A replication defective recombinant Ad5 vaccine expressing Ebola virus GP is safe and immunogenic in healthy adults. Vaccine (2010) 29:304–13. doi:10.1016/j.vaccine.2010.10.037
