# Supplementary material for: Herd Immunity to Ebolaviruses Is Not a Realistic Target for Current Vaccination Strategies
Source: Front Immunol. 2018 May 9;9:1025. doi: 10.3389/fimmu.2018.01025 (PMC5954026; doi:10.3389/fimmu.2018.01025)
Supplement: Supplementary file 5 [file Data_Sheet_5.PDF]

Data Sheet 5. Populations and percentage of rural populations of African countries that have been affected by Ebolavirus outbreaks.

Country	Population	% Rural population
Côte d'Ivoire	23,254,184[1]	46[1]
Democratic Republic Congo	79,722,624	58
Gabon	1,763,142	13
Guinea	12,947,122	63
Liberia	4,615,222	50
Mali	18,134,835	60
Nigeria	186,987,563	52
Republic of the Congo	4,740,992	35
Senegal	15,589,485	56
Sierra Leone	6,592,102	60
South Africa	54,978,907	35
South Sudan	12,733,427	81
Uganda	40,322,768	84
	**Total: 462,382,373**	

[1] Data derived from the world bank website (http://www.worldbank.org, last accessed on 17th October 2017)
